# Computer Based Assessment (CBA): Perception of residents at Dow University of Health Sciences

**DOI:** 10.12669/pjms.304.5444

**Published:** 2014

**Authors:** Masood Jawaid, Foad Ali Moosa, Farhat Jaleel, Junaid Ashraf

**Affiliations:** 1 Dr. Masood Jawaid, MCPS, MRCS, FCPS, Assistant Professor Surgery, Incharge e-Learning at Dow University of Health Sciences, Dow University Hospital and Dow International Medical College, Dow University of Health Sciences, Karachi, Pakistan.; 2 Prof. Foad Ali Moosa, FRCS, Professor of Surgery, Dow University Hospital and Dow International Medical College, Dow University of Health Sciences, Karachi, Pakistan.; 3 Dr. Farhat Jaleel, FCPS, Associate Professor of Surgery, Dow University Hospital and Dow International Medical College, Dow University of Health Sciences, Karachi, Pakistan.; 4 Prof. Junaid Ashraf, FRCS, Chairman Postgraduate Committee, Principal and Professor of Neurosurgery, Dow Medical College, Dow University of Health Sciences, Karachi, Pakistan.

**Keywords:** Computer based assessment, Formative assessment, e-assessment

## Abstract

***Background and objective*** ***: ***During the past few years, Computer-based assessment (CBA) has gained popularkity as a testing modality. This assessment offers several advantages over paper based assessment (PBA) testing. The objective of this study was to find out residents’ perception of this method of assessment.

***Methods*** ***: ***The post graduate residents of Dow University of Health Sciences in the field of Surgery, Medicine, Gynecology and Obstetrics experienced their first formative Computer-based assessment (CBA) in year 2013.Immediately after formative CBA, an anonymous paper based questionnaire was distributed amongst the residents and response was sought for their self-perceived computer usage competence before starting residency, perceptions regarding CBA method and to determine their preference for PBA or CBA in future assessment preferences.

***Results: ***Total 173 residents completed the questionnaire. More than half of residents, 56.1% had no prior experience of CBA. Three fourth, 76.4% of the residents were less than confident before sitting in CBA, while after completing CBA, 64.8% were either confident or extremely confident for CBA. Most common problem encountered by students was logging in 28.9%. More students (53.2%) believed that paper assessment took longer to complete than CBA. Majority of the students (61.8%) rated CBA as better than PBA despite experiencing it for the first time.

***Conclusion: ***Resident’s perception for CBA is good and they recommend its use in future assessment as well. However, to take maximal advantage of this technology, faculty should be trained to develop questions not only with text and pictures but with audio and video support.

## INTRODUCTION

During the past few years, Computer-based assessment (CBA) has gained popularity as an assessment modality.^1^Tests can be taken independent of time or physical place and a unique test for each individual candidate can be generated with same difficulty index. Tests can be scored immediately after, providing instant, individualized and detailed feedback for each candidate. Another major advantage of CBA is that it can make use of multi-media materials (video, audio) in test items,^2^ thus increasing the validity of the test. With this modality, individual performance of candidates can be compared against group performance. All these features enable CBA highly suitable for use in official high stake examinations.^3^

However, there are also some disadvantages, among which is the very high initial cost of CBA setup and also this mode is not suitable for every type of assessment (example: extended response questions, performance). Other disadvantages are computer glitches, content errors, computers and server crashes and data security lapses.^4^Some students are used to taking notes in question paper and also in a habit of marking questions and/or answers for later review which is not very much suitable with this system. Some students read more quickly and more easily on paper than on a glaring computer screen.

From the students’ perspective of the CBA, there have been varied perceptions. One study reported that more students anticipated problems with the CBA than actually experienced them.^5^Before completing the assessment, fewer students were confident about CBA, however more students stated a preference for CBA after their first experience.^3^Studies conducted have showed a trend of preference for CBA over PBA.^6^ Some studies reported the main disadvantage as being increased anxiety amongst those inexperienced with use of computer^3^^,^^5^ and regarded them as “technophobic”.

However, the transition to CBT is neither easy nor cheap. Dow University of Health Sciences (DUHS),being one of the largest universities of public sector in Pakistan is working on innovations in the field of medical education.^7^ Currently Dow University of Health Sciences is using pen and paper method for the assessment of student`s knowledge. In the past few years, the number of programs offered by the University as well as the number of students has increased significantly and the conventional testing method has become time consuming and difficult to administer. We are taking advantage of the fact that all institutes of the University are well equipped with Information Technology (IT) department and have state of the art digital libraries. More ever now e-Learning Cell in the university is also functional. Utilizing these resources, a solution of examination in large classes of students, CBA was planned to be explored.

Before introducing CBA in high stake tests, it is important to find out student perception and also how they compare CBA and PBA. Experience gained with this exercise will be helpful for administration, faculties and students to work for high stake summative assessment with this modality.

## METHODS

Every year post graduate residents of DUHS are required to sit in formative assessment by pen and paper method. During the year 2013, all residents working in Surgery, Medicine, and Gynecology and Obstetrics appearing in formative assessment were assessed first time with CBA modality in Digital Library.

Dow University of Health Sciences was already equipped with locally hosted Learning Management System, MOODLE so the quiz activity of the software^8^ was used to administer the assessment which include 100 item one best multiple choice questions. Question for all students were same but sequence was different for every student and also sequence of options was different by using software technology. The test time was two hours. As this modality was utilized for the first time; students were given demonstration of usage of the software for approximately five minutes before the test. Students then went through a practice test so that they could familiarize themselves with the workflow of software, editing answers once marked and different navigational features.

Assessment test was taken by 206 residents. Immediately after the test, student perceptions of the assessment methods were enquired by a paper based self-administered questionnaire. Intentionally this method was used as those students who were not satisfied with this assessment method or found difficulty with computers during test, could give a feedback easily. Questionnaire also gathers information about demographic profile, resident’s self-rated competence in using computer and comparisons of their opinion about CBA and PBA. Data was analyzed with SPSS 17. Descriptive statistics was used to present data.

## RESULTS

A total of 206 residents took the test. Out of these 173 residents completed and returned back the questionnaire. These included 58 (33.5) surgical, 67 (38.7%) Gynecology and Obstetrics and 48 (27.7%) Medicine residents. The response was 83.9%. There were 114 females (65.9%) and 59 males (34.1%).These included first year residents which were 60 in number (34.7%), second year resident 50 (28.9%), third year 31 (17.9%) and final year residents were 28 (16.2%). 

Responding to their competence about use of computer prior to entering residency program, more than 60%rated themselves as at least “competent” in using e-mail and the internet. One fourth of the students had no prior experience of spread sheets. It is also important to note that 6.4% had never used email prior to starting residency (Table-I). Majority (68.2%) rated their overall computer competence as “adequate” or “more than adequate” before starting residency training.

More than half of residents, (56.1%) had no prior experience of CBA. Training (demonstration of the software usage) was found to be at least “adequate” by more than 80% of residents. Three fourth 76.4% of the residents were less than confident before sitting in CBA, while after finishing their CBA, 64.8% were either confident or extremely confident. Most common problem experienced by the residents was of login issue in (28.9%), followed by saving and editing answers in (9.2%). Most (64.3%) of the residents reported no problems (Table-II).

More than half of residents (53.2%) said that PBA takes longer to complete than CBA, whereas 15% thought the opposite. Majority of the students (61.8%) rated CBA as better than PBA despite majority had attempted it for the first time. When asked which method of assessment they would prefer to use in future, 67.1% responded with CBA, 9.8% preferred PBA and 16.8% responded that they had no preference (Table-III).

Few representative open remarks of residents were:

*“It was nice exposure to have online test first time for postgraduate trainees. Hope we will have same in future too”*

*“* *I appreciate this attempt of examination and recommend it to be taken half-yearly rather annually. I also recommend that DUHS should provide awards/certificates/prizes to the candidates who score the highest marks in order to have feeling of appreciation and have serious and worthy response from trainees.”*

## DISCUSSION

The findings of this study showed that CBA was very well received by the residents, despite the fact that, for most of them this was the first experience of its kind. No major technical problems were encountered apart from few which were rectified at the same time. Residents preferred CBA compared to PBA and requested that this method of assessment should be utilized more frequently instead of being carried out only on yearly basis. 

**Table-I:** Competence in using computer prior to entering Residency

	***Never used***	***Less than competent***	***Competent***	***More than competent***	***Total***
Email	11 (6.4%)	44 (25.4%)	108 (62.4%)	10 (5.8%)	173 (100%)
Internet	8 (4.6%)	48 (27.7%)	105 (60.7%)	12 (6.9%)	173 (100%)
Word Processing	17 (9.8%)	61 (35.3%)	89 (51.4%)	4 (2.3%)	173 (100%)
Spreadsheets	45 (26.0%)	74 (42.8%)	49 (28.3%)	2 (1.2%)	173 (100%)
Computer/ Mobile Games	9 (5.2%)	51 (29.5%)	97 (56.1%)	14 (8.1%)	171 (98.8%)

**Table-II:** Feedback of residents about Computer Based Assessment (n = 173).

***Question***	***n (%)***
*Previous experience of submitting work online for assessment*	
Yes No	75 (43.4) 97 (56.1)
*Rating for the guidance, instructions and demonstrations used for training this assessment software*	
Less than adequate Adequate More than adequate	26 (15.0) 126 (72.8) 18 (10.4)
*Prior to using this system, rating of in the online system as a method of assessment.*	
Not confident Somewhat confident Confident Extremely confident	38(22.0%) 93(54.4%) 32(18.7%) 7(4.0%)
*After CBA, rating of in the online system as a method of assessment.*	
Not confident Somewhat confident Confident Extremely confident	10 (5.8) 49 (28.6) 102 (59.6) 9 (5.2)
*Limitations that were encountered when using the online test.[Total resident encountered problem = 61 (35.6%)] * *Few of them encounter multiple issues*	
Accessing the software Security of the system Problems with logging in Submitting your answers Saving and editing your answers Others	15 (8.7%) 12 (6.9%) 50 (28.9%) 7 (4.0%) 16 (9.2%) 3 (1.7%)

* All residents not responded to all questions so total percentage is not 100%

**Table-III:** Resident’s comparison of Computer Based Assessment and Paper Based Assessment

***Method of assessment that took longer to complete.***	
Online Test Paper assessment No difference	26 (15.0%) 92 (53.2%) 45 (26.0%)
*Rating for online test compared with the paper based assessment.*	
Online test better Paper assessment better No difference	107 (61.8%) 11 (6.4%) 43 (24.9%)
*If had a choice, preference to use online or paper-based assessment*	
Online test Paper assessment No preference	116 (67.1%) 17 (9.8%) 29 (16.8%)

Results of this study were not different from that of other studies about CBA. A study among physiology students reported that students are satisfied with CBA.^9^ More than half of the students (52%) rated CBA as better than PBA. Students said this method takes shorter time to complete. When asked which mode of assessment they would prefer in future, 53% opted for CBA. Another study among final year undergraduate students reported that 79.8% preferred CBA compared to PBA.^3^ Reasons for preference included they could proceed at their own pace, examination was fun, convenient and there was equality in marking.

CBA is not a method of improving the item weakness. Validity and good construction of items is always a pre requisite for a good assessment. This can be highlighted by a study among students of undergraduate chemistry.^10^ Only 29.2% were in favor of CBA. When reasons were probed, findings were that it was due to flawed chemical formulas, equations and structures in the test items.^10^

The implementation of CBA is not just transferring traditional testing models from paper to screen. The main purpose of using technology in assessment is to improve the quality of assessment in terms of validity and efficiency.^11^Incorporating the latest technological developments into testing requires significant research and investment in fields such as multimedia design, network issues, items quality and security, psychometrics, and information technology. All of these need to be addressed before embarking CBA as a regular feature in institutes.^12^

Future work is required for assessing test psychometric of CBA and comparing them with PBA, faculty perceptions especially after they have developed test items on multimedia format, as currently we have only used a few pictures in the test. Good formative assessments should result in increased student’s performance in summative assessments. For this, these areas need to be explored in future.

## CONCLUSION

Residents rated CBA highly; despite the fact that for most of them, it was their first experience. Few technical issues were encountered during assessment, but all of them were resolved without any major issue. CBA offers a range of advantages: place-independent assessment in formative test, the use of multimedia, the automatic processing of results, automatic statistical analysis of exams and the reusability of questions. This makes this mode of assessment a highly efficient way of assessment for large number of students.

## Notes

***Source of funding: ***None.
